# Need for concomitant Akin osteotomy in patients undergoing Chevron osteotomy can be determined preoperatively: a retrospective comparative study of 859 cases

**DOI:** 10.1186/s13018-019-1319-2

**Published:** 2019-08-28

**Authors:** Gerhard Kaufmann, Maximilian Hofmann, Matthias Braito, Hanno Ulmer, Alexander Brunner, Dietmar Dammerer

**Affiliations:** 1 OFZ Innsbruck, Innrain 2/3, Stock, 6020 Innsbruck, Austria; 2 Department of Orthopaedic Surgery, Medical University of Innsbruck, Anichstrasse 35, 6020 Innsbruck, Austria; 3 Department of Medical Statistics, Informatics and Health Economics, Medical University of Innsbruck, Anichstrasse 35, 6020 Innsbruck, Austria

**Keywords:** Hallux valgus, Radiological outcome, Chevron osteotomy, Akin osteotomy

## Abstract

**Background:**

The Chevron osteotomy is a frequently used surgical method for hallux valgus correction. This method is often combined with an Akin osteotomy. To date, clear guidelines for the implementation of an additional Akin osteotomy are missing. The purpose of this study was to elucidate the impact of concomitant phalangeal correction on the outcome after hallux valgus surgery and to define indication criteria for an additional Akin osteotomy.

**Methods:**

Patients (859 feet) undergoing distal Chevron osteotomy at our department were retrospectively grouped into group C (Chevron, 785 feet) and group AC (Chevron plus Akin, 74 ft). Radiological assessment including the intermetatarsal angle (IMA), the hallux valgus angle (HVA), the distal metatarsal articular angle (DMAA), and the proximal to distal phalangeal articular angle (PDPAA) was performed preoperatively, postoperatively, after 6 weeks, and after 3 months. Longer-term follow-up with a mean of 36.4 months was available for 248 cases (29%).

**Results:**

A significant improvement of all parameters could be found to all points of survey (*p* < 0.001). Loss of correction was detected for HVA (*p* < 0.001) and IMA (*p* < 0.007) with higher levels in group C. Preoperative PDPAA exceeding 8° correlated significantly with loss of HVA correction in group C (*p* < 0.001).

**Conclusion:**

The combined Chevron and Akin osteotomy allowed for better correction of the hallux valgus deformity with better maintenance of the achieved correction. Recommendation for concomitant Akin osteotomy may be determined by a preoperative PDPAA exceeding 8°.

**Trial registration:**

Retrospectively registered. UN5080.

**Level of evidence:**

Therapeutic, Level III, retrospective comparative series.

## Background

Distal Chevron osteotomy is predominantly used for mild to moderate hallux valgus deformity and has become one of the most frequently performed surgical techniques to correct hallux valgus deformity [1–3]. A variety of other surgical techniques are used for mild to moderate hallux valgus deformities as well [4]. The question which method reveals the best clinical and radiological outcome is still discussed controversially [5]. Although good clinical outcome can be observed with different surgical techniques [6], loss of correction can be found frequently from the initial to the radiograph after 12 weeks [7]. In accordance to this, recurrence is a generally observed situation after hallux valgus correction [8, 9]. Recently, some studies have been published focusing on the identification of outcome predicting factors [10, 11]. However, the role of hallux valgus interphalangeus and its correction by means of an additional Akin osteotomy is discussed controversially to date [12–15]. In a recently published paper, it has been shown that hallux valgus interphalangeus is part of the total deformity, especially in mild cases [13]. Whereas excellent radiographic correction of the deformity has been detected immediately after bunion correction in combination with proximal phalangeal Akin osteotomy [15], and midterm outcome showed moderate superiority only [10]. However, maintenance of the achieved correction is variable. Therefore, the value of an additional Akin osteotomy for correction of hallux valgus deformity remains uncertain.

The absence of clear guidelines for additional phalangeal osteotomy in respect to the preoperative radiograph makes the application of an Akin osteotomy a surgeon’s decision to date. After an isolated metatarsal correction, a significant increase of radiographic hallux valgus interphalangeus has been found intraoperatively [16] with an average deterioration of 2° for PDPAA from pre- to postoperative [17]. Recently, a preoperative PDPAA of 10° has been recommended to be a possible cutoff value for additional Akin osteotomy [18], but the underestimation of a hallux valgus interphalangeus deformity on preoperative films seems likely. Several authors propose an additional Akin osteotomy depending on the intraoperative status after metatarsal correction [16, 19]. In respect of precise deformity planning, the determination of specific preoperative radiological parameters requiring an additional Akin osteotomy would be useful.

The purpose of our radiological study was to assess the contribution of Akin osteotomy on outcome after distal Chevron osteotomy. The second aim of this study was to evaluate if a specific preoperative cutoff value for PDPAA necessitating concomitant Akin osteotomy can be determined.

## Methods

### Patient selection

This study was in accordance with the ethical standards outlined in the 1964 World Medical Association Declaration of Helsinki and its later amendments following the Consolidated Standards of Reporting Trials (CONSORT) guidelines. The study was approved by the local ethics committee. The study subjects were adult patients with symptomatic idiopathic hallux valgus deformity. Only patients with isolated Chevron or Chevron with concomitant Akin osteotomy with complete radiological follow-up were included in this study. Data was collected retrospectively from patients, who have had surgery by multiple surgeons between January 2002 and December 2012 at our department via electronic search by means of the ICD (International Statistical Classification of Diseases and Related Health Problems, WHO) and the MEL-code (benefit-related coding system in national hospitals, National Ministry of Health). In regard to the inability of the electronic search to select different types of metatarsal osteotomies, the applied surgical method of every listed patient was verified by controlling the medical chart. Patients with proximal or midshaft osteotomies were not assigned to the study. Patients with additional surgeries of the lesser rays (e.g., Weil osteotomy, proximal metatarsal osteotomies of the lesser rays, hammertoe operation) were excluded to prevent secondary effects on the assessed radiological parameters following changes of the metatarsal anatomy. Only patients having undergone surgery because of a hallux valgus deformity were included, following the definition of a hallux valgus deformity with a preoperative HVA of more than 20° or an IMA of more than 10°. According to our institutional guidelines, an additional Akin osteotomy was performed in case of a hallux valgus interphalangeus with a PDPAA greater than 15° or if the hallux valgus deformity surmounted 10° after completing the Chevron osteotomy and the soft-tissue release. None of the participating surgeons considered an Akin osteotomy to be mandatory in every hallux valgus case. Despite the institutional agreement listed above, the application of an additional Akin osteotomy remained a surgeon´s decision. Shortening Chevron osteotomies because of osteoarthritic changes of the metatarsophalangeal joint as well as dorsal closing wedge Chevrons were not in our focus and could be excluded by means of the electronic searching terms. To exclude juvenile hallux deformity, patients under the age of 18 years were not assigned to the study as well.

Radiographic analysis was performed preoperatively, the first day postoperative, and 6 weeks and 12 weeks postoperatively as part of our clinical routine in hallux valgus patients. For all patients, an additional survey of the medical chart was made for further controls beyond the clinical routine. In every patient, the latest available radiographs were used for analysis only. We determined 6 months to be the minimum follow-up time for inclusion for midterm follow-up. Radiological assessment was made irrespective to the reason, why patients continued to present for care beyond the typical postoperative period. From the patients without clinical or radiological follow-up, information about midterm and long-term outcome is lacking. Seven cases were excluded due to early revision because of hardware failure or fracture. Five cases were excluded because of conversion to a fusion of the greater toe joint. Besides the seven cases excluded because of early hardware failure and the cases having undergone conversion to fusion, we did not find reports of surgical revisions so far.

**Fig. 1:**
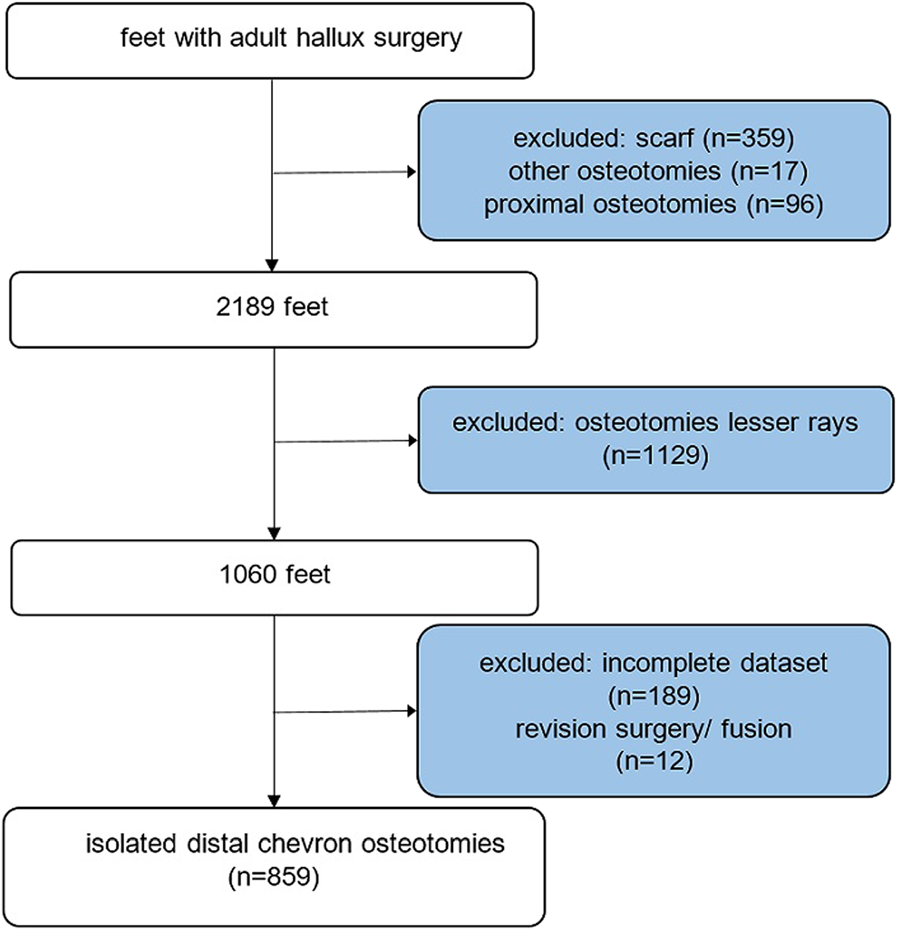
Flowchart diagram of data recruitment

In total, we identified 859 ft, which met the inclusion criteria. Mid- to long-term follow-up radiographs were available for 248 cases (29%), 218 in group C (28%) and 30 (41%) in the AC group. The mean follow-up time was 36.4 months on the average (SD of 41.3 months; shortest follow-up of 6 months in three cases). Both the high variance of follow-up times and the high drop-out rate are ascribed to the fact that patients have not been scheduled for follow-up examinations beyond 12 weeks postoperatively. Further detailed information of data recruitment is listed in a flowchart diagram (Fig. 1).

### Radiographic evaluation

**Fig. 2:**
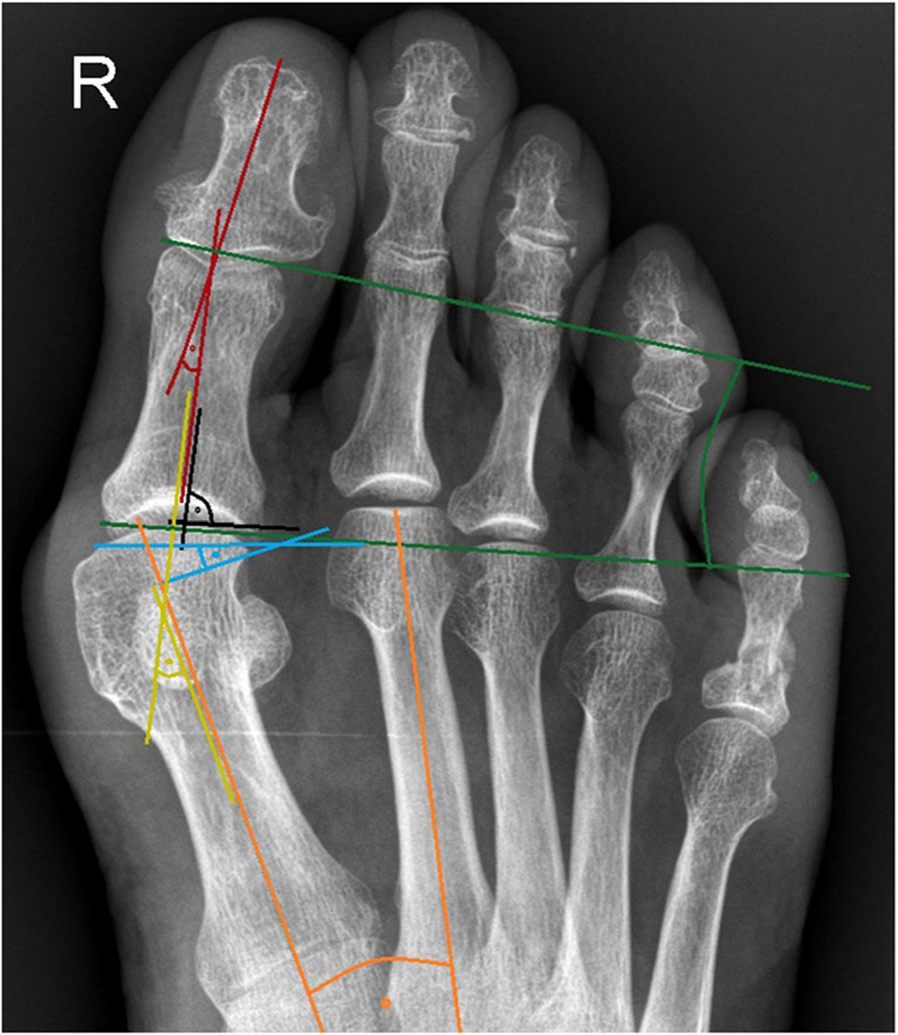
Anteroposterior radiograph showing all relevant angles used to define deformity: HVA (yellow lines), IMA (orange lines), DMAA (blue lines), and PDPAA (green lines)

All anteroposterior and lateral radiographs were taken in a standardized manner with the patient in a standing position. Radiographs were analyzed digitally using the Icoview software (syngo.share, ITH icoserve healthcare GmbH, Siemens) by trained foot and ankle fellows advised by an orthopedic surgeon, who was not involved in the patients’ care. The following radiographic outcome measurements (Fig. 2) were used in this study as have been described before [12, 20]: (1) the hallux valgus angle (HVA), defined as the angle between the midshaft axis of the first metatarsal and the proximal phalanx on the standing anteroposterior radiograph; (2) the intermetatarsal angle (IMA), an angle between the intersecting midshaft longitudinal axis of the first and second metatarsal on the standing anteroposterior radiograph. In the postoperative radiographs, it was measured as the angle subtended by the lines from the center head to the center base of the first and second metatarsal; (3) the distal metatarsal articular angle (DMAA), which is formed by a line perpendicular to the longitudinal axis of the first metatarsal and a line running through the edges of the articular surface of the head of the first metatarsal on the standing anteroposterior radiograph. By definition, a positive value for the DMAA is represented by a valgus tilt of the articular surface in relation to the axis of the metatarsal bone; (4) the proximal to distal phalangeal articular angle (PDPAA), which is assembled by the proximal and the distal joint line of the proximal phalangeal bone of the greater toe; (5) the position of the tibial sesamoid in relation to the midshaft axis of the first metatarsal (seven-part grading system) and (6) the joint congruity of the greater toe joint, which was expressed as the angle assembled by the joint lines of the metatarsal head and the proximal joint line of the proximal phalangeal bone.

Loss of correction was correlated in regard to the preoperative PDPAA and the application of an additional Akin osteotomy. Due to a malprojection with standard views, a deterioration of 2° for PDPAA after metatarsal correction can be expected preoperative [17]. In regard to this finding, we presumed a preoperative PDPAA of 8° to be more specific than the previously proposed cutoff value of 10° [18].

### Surgical technique

The exact operative technique of the Chevron osteotomy and the postoperative treatment have been previously described [21]. The distal soft-tissue procedure was performed in all cases either through the 4-cm dorsomedial incision made for the osteotomy or via a separate dorsolateral incision. The transverse intermetatarsal ligament was released, and a T-shaped capsulotomy of the lateral joint capsule was performed to allow for the repositioning of the sesamoids. If an Akin procedure was performed, the dorsomedial incision was lengthened to the proximal phalangeal bone. Protecting the flexor and extensor tendon, a horizontal v-shaped osteotomy was performed with the removal of the wedge. The lateral cortical bone was left intact to maintain a stable hinge. Two drill holes with a diameter of 1.5 mm were placed next to the osteotomy site. Closing of the osteotomy was performed after tunneling a No.2 polyglactin 910 suture (Vicryl, Ethicon, Johnson & Johnson) through the drill holes. After tying the sutures, the skin was closed with nylon No. 3 sutures.

Postoperative management was standardized in both groups. To maintain the positioning of the greater toe, soft dressings were applied. Patients were mobilized immediately in a custom-made hallux valgus shoe (Ofa GmbH, Bamberg, Germany). The surgical shoe was discarded after 6 weeks. Reduced weight-bearing was recommended for 2 weeks, followed by progressive full weight-bearing for 4 to 6 weeks.

### Statistical method

Sample characteristics are given as means, standard deviations, and frequencies. Comparisons of the Chevron and Chevron with Akin groups with regard to sociodemographic and clinical variables were based on Fisher’s exact tests and *t* tests for independent samples or Mann-Whitney *U* tests when data differs from the normal distribution. For pairwise comparisons between groups (Chevron versus Chevron with Akin) and within time frames (preoperative, postoperative, and follow-up) with regard to the radiographic outcomes, we used *t* tests for independent samples or Mann-Whitney *U* tests and paired *t* tests or Wilcoxon tests. In addition, we used linear regression analyses to describe associations between various radiographic angles. All statistical analyses were conducted with SPSS 20.0 (International Business Machines Corporation, Armonk, NY, USA).

## Results

A total of 859 ft with distal Chevron osteotomy met inclusion criteria for this survey (Fig. 1). In 74 ft (9% of cases), Chevron osteotomy was accompanied by an Akin osteotomy (AC group), while isolated Chevron osteotomy (C group) was performed in 785 ft (91%). Table 1 summarizes patient demographics for both groups. The groups showed no significant differences with regard to the distribution of sexes (*p* = 0.222), 9.7% of the cohort were male (83 ft). The average age at the time of surgery was 52.2 years (SD 14.5) with no difference between the two groups (*p* = 0.984). Baseline radiological data is summarized in Table 2 and was comparable between the two groups, with one exception in terms of the PDPAA.

**Table 1:** Baseline characteristics of cohorts C and AC

Variable		C Group (*n*=785)	AC Group (*n*=74)	Sig.
Male		73 (9.3%)	10 (13.5%)	.222^a^
Female		712 (90.7%)	64 (86.5%)	.222^a^
Age (years)*		52.2 ± 14.5 (20.3–89.1)	52.2 ± 15.1 (21.1–81.8)	.984^b^
Right		392 (49.9%)	41 (55.4%)	.999^a^
Left		393 (50.1%)	33 (44.6%)	.999^a^

The values are given as the number of patients, with the percentage in parentheses

*C group* Chevron group, *AC group* Chevron and Akin group, *Sig.* significance

^a^Fisher’s exact test

^b^Two-tailed, non-parametric Mann-Whitney *U* test

*The values are given as the mean and the standard deviation, with the range in parentheses

**Table 2:** Preoperative radiological data for cohorts C and AC

	Chevron (C)		Chevron and Akin (AC)		Significance between C and AC
	Mean	SD	Mean	SD	*p* value
IMA	13.0	2.6	13.0	2.2	0.981
HVA	28.3	7.3	28.1	6.7	0.879
DMAA	12.5	7.4	10.3	6.9	0.015
PDPAA	6.7	3.9	10.1	4.6	0.000
Joint congruity	13.0	8.3	13.6	9.2	0.591
Sesamoid position	5.2	1.3	4.9	1.4	0.051

*IMA* intermetatarsal angle, *HVA* hallux valgus angle, *DMAA* distal metataarsal articular angle, *PDPAA* proximal to distal phalangeal articular angle, *SD* standard deviation

We could detect significant improvement of HVA, IMA, joint congruity, and the positioning of the sesamoids in both groups from pre- to postoperative and to all points of survey (*p* < 0.001). We found a significant correction of HVA from 28.3 (SD 7.3) to 9.6 (SD 5.6) degrees in the C cohort and from 28.1 (SD 6.7) to 9.6 (SD 5.6) degrees in the AC cohort (*p* < 0.001).

**Fig. 3:**
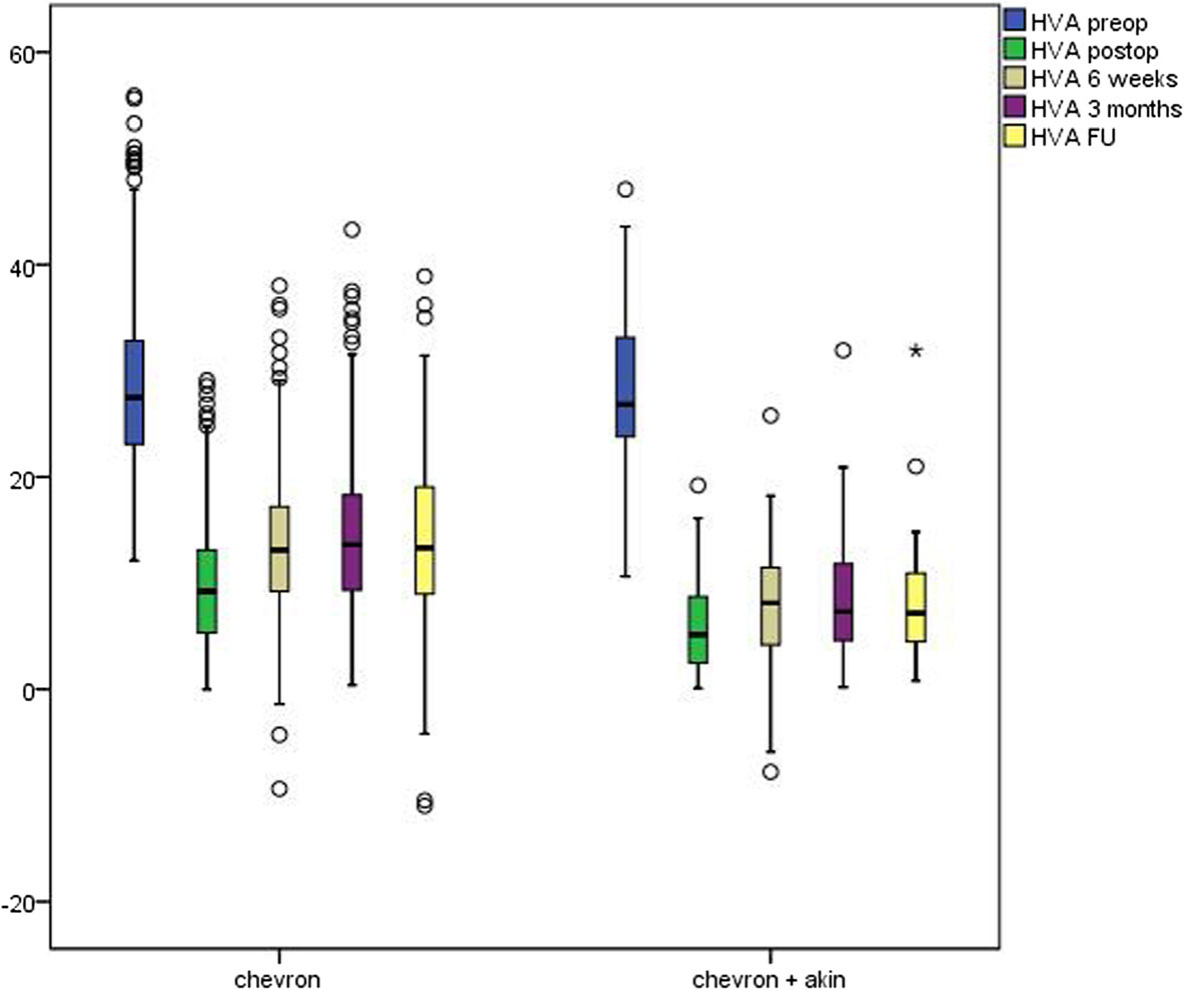
HVA (hallux valgus angle) pre- and postoperatively until follow-up for the Chevron and the Chevron/Akin cohort*. L*egends: HVA—vertical axis, C and AC groups—horizontal axis. The line inside the boxes represents medians, and the whiskers represent maximum and minimum 1.5 interquartile range. HVA preop: hallux valgus angle preoperative. HVA postop: hallux valgus angle postoperative. HVA 6 weeks: hallux valgus angle after 6 weeks. HVA 3 months: hallux valgus angle after 3 months. HVA FU: hallux valgus angle at follow-up

Improvement of HVA was better to all points of survey in the AC than in the C cohort. Throughout follow-up, HVA showed a significant deterioration in group C but not in group AC (Fig. 3). After 3 months, HVA amounted 13.9 (SD 6.7) in the C and 8.6 (SD 5.7) degrees in the AC cohort. In the group with available films at follow-up beyond 3 months (248 cases), we found 55 cases with radiographic recurrence in terms of HVA bigger than 20 or IMA surmounting 10° (3 in the AC group and 52 in the C group), the majority of them remaining subclinical.

Superiority of the AC cohort could be found for the other parameters as well. Table 3 shows all measured parameters from postoperative to follow-up and intergroup correlation for both groups. For IMA and positioning of the sesamoids, we could detect moderate loss of correction close to significance in both groups. Until follow-up, no further deterioration could be found. The improvement of DMAA in both groups lacked significance to all points of survey with negligible changes throughout follow-up. Obviously, significant correction of PDPAA could be determined exclusively in group AC (*p* < 0.001). This angle stayed stable in both cohorts with negligible changes throughout follow-up.

**Table 3:** Postoperative radiological data for cohorts C and AC

	Chevron (C)		Chevron/Akin (AC)		Significance between C and AC
	Mean	SD	Mean	SD	*p* value
IMA postoperative	4.6	2.4	4.1	2.4	0.109
IMA 6 weeks	5.9	2.8	5.1	2.9	0.026
IMA 12 weeks	6.4	3.0	6.0	3.2	0.339
IMA FU	6.0	2.8	4.5	2.9	0.007
DMAA postoperative	7.2	4.9	6.1	3.7	0.087
DMAA 6 weeks	6.7	6.8	4.9	7.7	0.053
DMAA 12 weeks	7.6	5.6	6.9	5.3	0.314
DMAA FU	7.4	6.7	6.1	7.8	0.333
PDPAA postoperative	7.8	3.8	5.5	2.9	0.000
PDPAA 6 weeks	7.7	4.3	4.3	3.2	0.000
PDPAA 12 weeks	7.5	3.8	6.0	3.8	0.001
PDPAA FU	7.0	4.0	4.5	2.3	0.001
Joint congruity postoperative	5.2	5.1	5.5	5.0	0.706
Joint congruity 6 weeks	6.2	6.2	8.4	7.6	0.008
Joint congruity 12 weeks	5.7	5.5	7.0	6.6	0.053
Joint congruity FU	6.6	6.9	9.2	6.0	0.049
Sesamoids postoperative	1.6	0.8	1.4	0.6	0.048
Sesamoids 6 weeks	2.3	0.9	2.1	1.1	0.064
Sesamoids 12 weeks	2.2	1.2	1.9	1.0	0.058
Sesamoids FU	2.7	1.1	2.1	0.9	0.004

*IMA* intermetatarsal angle, *DMAA* distal metatarsal articular angle, *PDPAA* proximal to distal phalangeal articular angle, *SD* standard deviation, and *FU* follow-up

**Fig. 4:**
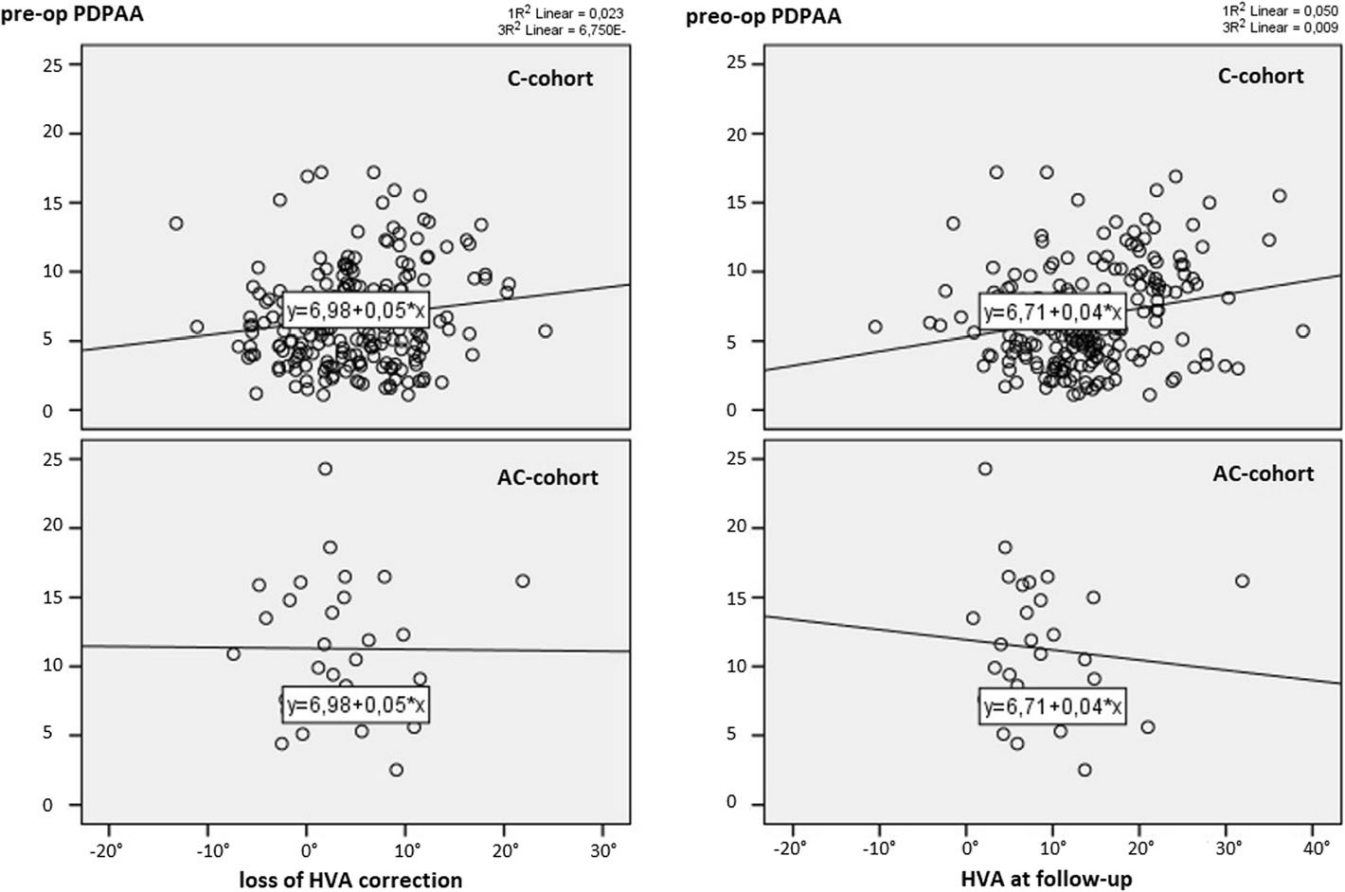
Scatterplot showing correlation of preoperative PDPAA in degrees (vertical axis) and loss of correction of HVA in degrees (left figure) and HVA at follow-up in degrees (right figure)

To evaluate a possible influence of the preoperative PDPAA on outcome in terms of loss of HVA correction and HVA at follow-up, a scatterplot analysis was made. We could detect a direct correlation of preoperative PDPAA with loss of HVA correction as well as with HVA at follow-up (Fig. 4). Further analysis revealed significantly higher HVA levels at follow-up in the C subgroup exceeding 8° of preoperative PDPAA (*p* < 0.001) (Table 4). Loss of HVA correction from postoperative to follow-up was 3.2° in the subgroup below and 6.8° in the subgroup above the preoperative PDPAA cutoff value in the C group. In the AC group, loss of HVA correction amounted to 3.2 and 2.3°.

**Table 4:** Correlation of preoperative PDPAA and HVA for cohort C and AC

	PDPAA < 8°		PDPAA > 8°		
	Mean	SD	Mean	SD	*p* value
HVA preoperative (C)	28.0	6.9	28.7	8.1	0.221
HVA postoperative (C)	9.3	5.4	10.2	5.8	0.038
HVA 6 weeks (C)	12.8	5.4	14.7	7.2	0.001
HVA 12 weeks (C)	13.5	6.4	15.0	7.4	0.006
HVA FU (C)	12.5	6.8	17.0	8.3	0.000
HVA preoperative (AC)	29.5	6.7	27.3	6.7	0.196
HVA postoperative (AC)	6.0	3.8	6.2	4.8	0.818
HVA 6 weeks (AC)	9.7	6.1	7.8	5.6	0.219
HVA 12 weeks (AC)	8.4	5.0	8.9	6.3	0.710
HVA FU (AC)	9.2	6.0	8.5	6.7	0.811

*HVA* hallux valgus angle, *PDPAA* proximal to distal phalangeal articular angle, *SD* standard deviation, *FU* follow-up, *AC* Chevron and concomitant Akin, *C* Chevron

## Discussion

An important finding of our study was that the radiological outcome after hallux valgus correction with Chevron and concomitant Akin osteotomy was superior compared to the isolated Chevron procedure. An additional Akin osteotomy was performed in about 10% of cases (74 ft from a total of 859 ft). The different sizes of the two group (AC and C) result from individual decision making for the application of an additional Akin procedure. HVA correction in cohort AC was markedly superior with a reduced loss of correction throughout follow-up. This finding may be ascribed to the methodology of the Akin osteotomy, since it directly affects the phalangeal anatomy. A second and probably the most substantial finding of this survey was the identification of a preoperative PDPAA threshold value of 8° with influence on loss of HVA correction. This detection was supported by a higher HVA at follow-up in cases surmounting this threshold value in the C group.

Preoperative deformity in our study population was comparable to published literature [22]. Our cohort experienced significant pre- to postoperative correction of the hallux valgus deformity to all points of survey, in terms of IMA, HVA, DMAA, joint congruity, and positioning of the sesamoids with superiority of the AC cohort. The mean correction at follow-up in our study population was 14.6° for HVA, 7.0 for IMA, and 5.1 for DMAA in the Chevron group and 19.5, 8.5, and 4.2 in the AC group. The achieved correction of both groups was higher, but still comparable to other studies [22, 23]. In regard to the fact that 81 cases of our study cohort had a moderate to severe deformity in terms of the IMA (> 16°), this finding might be a consequence of stretching the surgical Chevron technique to its technical limit.

Loss of correction for HVA and IMA could be found in the C and AC groups with higher amounts in the C cohort. We ascribe this detection to a better biomechanical restoration of the forefoot, if phalangeal pathology was addressed as well. Subgroup analysis of the groups with long-term follow-up (248 cases) and the group with 3 months results only (611 cases) showed comparable results after 3 months. The majority of loss of correction was found from postoperative to 6 weeks, while loss of correction from 3 months to follow-up was negligible. From postoperative to 6 weeks, the majority of loss of correction may be ascribed to the methodology of the immediate postoperative films. The majority of these radiographs are taken with partial weight-bearing. Tanaka et al. have reported a direct correlation of weight-bearing and radiological parameters on radiographs already [24]. Furthermore, radiographic angles used to determine hallux valgus deformity have been shown to be influenced by rotation and inclination of the hallux as well [25, 26]. Nevertheless, the postoperative radiograph remains mandatory and is an important tool for controlling surgical outcome after bunion correction. In this context, Park et al. could determine some parameters from the immediate postoperative radiograph, which may be used as outcome predicting parameters [27].

Interestingly, we found better correction of DMAA and the position of the sesamoids in the AC cohort as well. Given that in both cohorts the surgical technique of the Chevron osteotomy was identical and a lateral release was performed in every case, we attribute this finding to a better biomechanical restoration of the deformity. Nevertheless, for DMAA, a significant variation on the standardized radiographs have been shown already [26].

In literature, the role of additional Akin osteotomy in hallux surgery remains unclear [15, 17]. The group around Strydom identified hallux valgus interphalangeus as a common deformity contributing significantly to the total hallux deformity [13]. However, due to a pronational deformity of the greater toe in hallux valgus deformity, preoperative diagnosis of phalangeal pathology may be hampered. On possible solution would be to intraoperatively verify phalangeal pathology after metatarsal correction in moderate to severe hallux valgus cases [16]. In our study, we could find significantly better results in terms of HVA at final follow-up after Chevron osteotomy, if concomitant Akin osteotomy was performed in cases surmounting the PDPAA cutoff value of 8°. In cases of lower PDPAA values, phalangeal pathology seems to be negligible and the effect of additional Akin osteotomy remains limited, particularly given the risk of complications such as flexor tendon laceration, IP-joint stiffness, malcorrection, and non-union.

## Limitation

Limitations stem from the monocentric character, the retrospective, and the radiographic nature of this study. Other limitations are the high drop-out rate and the different sample sizes. Our standard clinical routine covers controls immediately after surgery, after 6 weeks, and after 12 weeks. Beyond this time frame, further controls occurred occasionally. This explains the short follow-up time of 36.4 months and the reduced number of patients available for follow-up examination (29%). However, since loss of correction from 3 months to follow-up was negligible, we consider our long-term cohort to be representative irrespective to the high drop-out rate at latest follow-up. We acknowledge the different sample sizes between the two groups, what may influence the observed results. Since the observed variances are comparable between the two groups, this should be of minor importance. The most positive aspect remains the size of our data pool.

## Conclusion

The combined Chevron and Akin osteotomy allowed for better correction of the hallux valgus deformity with better maintenance of the achieved correction. Recommendation for concomitant Akin osteotomy may be determined by a preoperative PDPAA exceeding 8°.

Authors’ contributions

Each author has contributed significantly to, and be willing to take public responsibility for, one or more aspects of the study. GK conceived of the study, generated its design, carried out parts of the measurements, and drafted the manuscript. MH carried out the measurements and is one of the co-authors. MB carried out the measurements and acts as the corresponding author. HU performed the statistical analysis and acts as a co-author. AB helped to draft the manuscript. DD participated in the study design and is a senior author. All authors read and approved the final submitted manuscript.

## Data Availability

All data generated or analyzed during this study are included in this published article. This study or contents of this study have not been published or submitted for publication elsewhere.

## Abbreviations

AC cohort: Akin and Chevron cohort
C cohort: Chevron cohort
DMAA: Distal metatarsal articular angle
HVA: Hallux valgus angle
IMA: Intermetatarsal angle,
PDPAA: Proximal to distal phalangeal articular angle
SD: Standard deviation
